# The implications of living with heart failure; the impact on everyday life, family support, co-morbidities and access to healthcare: a secondary qualitative analysis

**DOI:** 10.1186/s12875-016-0537-5

**Published:** 2016-09-26

**Authors:** Mirella Fry, Sarah McLachlan, Sarah Purdy, Tom Sanders, Umesh T. Kadam, Carolyn A. Chew-Graham

**Affiliations:** 1 Keele Medical School, Keele University, Keele, UK; 2 Department of Physiotherapy, Division of Health and social care Research, King’s College London, London, UK; 3 University of Bristol, Faculty of Health Sciences, Senate House, Tyndall Avenue, Bristol, UK; 4 University of Sheffield, School of Health and Related Research (ScHARR), Section of Public Health, Regent Court, Regent Street, Sheffield, UK; 5 Research Institute, Primary Care and Health Sciences, Keele University, Keele, UK; 6 Health Services Research Unit, Keele University, Keele, UK

## Abstract

**Background:**

The aim of this study was to use secondary analysis to interrogate a qualitative data set to explore the experiences of patients living with heart failure.

**Methods:**

The data-set comprised interviews with 11 patients who had participated in an ethnographic study of heart failure focusing on unplanned hospital admissions. Following an initial review of the literature, a framework was developed with which to interrogate the data-set. This was modified in light of analysis of the first two interviews, to focus on the rich data around patients’ perceptions of living with heart failure, managing co-morbidities, accessing healthcare and the role of their family and friends, during their illness journey.

**Results:**

Respondents described how the symptoms of heart failure impacted on their daily lives and how disruption of routine activity due to their symptoms caused them to seek medical care. Respondents disclosed the difficulties of living with other illnesses, in addition to their heart failure, particularly managing multiple and complex medication regimes and negotiating multiple appointments; all expressed a desire to return to their pre-morbid, more independent lives. Many respondents described uncertainty around diagnosis and delays in communication from their healthcare providers. The importance of family support was emphasised, but respondents worried about burdening relatives with their illness.

**Conclusion:**

Living with heart failure causes disruption to the lives of sufferers. Facilitation of access to healthcare, through good communication between services and having a strong support network of both family and clinicians can reduce the impact of heart failure on the lives of the patient and those around them.

## Background

A chronic illness, by definition, is a disease which persists for a long period of time and can cause continuous or episodic periods of incapacity [1]. Bury 1982 describes the effects of chronic illness as ‘biographical disruption’ to everyday life, and not only disruption to the individual suffering with the illness, but their families and wider social network [2]. Patients and their families may seek out information, support, and the most effective strategies to manage their symptoms, in the effort to minimise potential future disruption [3].

Heart failure is prevalent in the UK [4]; common symptoms include breathlessness, peripheral oedema and fatigue, all of which interfere with daily life [5]. Patients typically have multiple chronic co-morbidities, each of which may have a complex treatment regime [6]. Self-management of multiple chronic illnesses, educating patients to monitor their own health and being able to recognise illness severity are current features of healthcare policy [7].

Symptom unpredictability has been reported to leave patients feeling helpless and completely dependent on those around them, leading to a lack of control of the illness and an increased burden on family and the healthcare system [8]. Responsibility for care may shift from the patient to their spouse or immediate family. In these circumstances the patient may adopt the ‘sick role’, relying on their family for support both with their illness and previous responsibilities [9]. It has been reported [10] that in patients with greater levels of family support, confidence between members is increased, and family ties strengthened, in times of need such as ill-health. Therefore, congruent beliefs about the illness are important in order to achieve successful family functioning and to enhance patient well-being [10].

Previous studies exploring heart failure and patients’ quality of life suggest that balancing treatment regimens of multiple co-morbidities and the additional symptoms from co-morbidities could be extremely difficult for patients [5, 11]. Challenges for patients managing multiple illnesses include; lack of care co-ordination, a greater need for support and more time needed to develop techniques to manage multiple illnesses [9]. Patients’ understanding of heart failure and how to manage has been reported as poor, leading to a cycle of hospital (re) admissions, which is not only disruptive for the patient but also places significant pressure on secondary care and increases healthcare cost [5].

There appears to be little consideration to the interaction between different health conditions by healthcare professionals. However, patients have been reported to manage their multiple illnesses by active weighing up of possible treatment options across their different health conditions [11, 12]. The degree of agreement (or otherwise) in prioritising multiple conditions between the patient and clinician and between different clinicians and the patient, can affect subsequent self-management, such as treatment adherence and future therapeutic relationships [8].

The relationship between a patient and their doctor is the foundation for effective management of chronic illness [13, 14]. An effective doctor-patient relationship is founded on high quality communication, which could be defined as the promotion of information gathering, agreement on therapeutic options and patient support [15]. Effective organisation within the healthcare system can provide continuity within clinical relationships [16]. Conversely, poor communication can result in patient uncertainty, confusion and worry, reducing a patient’s quality of life and ability to recover from illness. In vulnerable patients the doctor-patient relationship is important as patients may experience increased dependence on the doctor [12, 17]. Effective communication can ensure that doctors are aware of the patients’ expectations, help to regulate their emotions and facilitate the understanding of information, which will ultimately lead to greater patient satisfaction [18]. Clinician and nurse availability can give patients a sense of security, as the patient understands that time and effort has been invested to accommodate them and provide information or reassurance [19].

This paper focuses on the secondary analysis of data collected from HoldFAST, a multicentre study including Bristol, Oxford and Keele Universities, which was funded by the National Institute for Health Research. The aim of collecting qualitative data from these participants was to gain an in-depth insight into the pathways towards, and reasons for, unplanned hospital admissions in patients with heart failure. The HoldFAST study aimed to explore patients’ experiences from multiple standpoints using ethnographic methods, combining observations, interviews and documentary data sources [20]. This paper reports a secondary analysis which focused on patients’ perspectives of living with and managing heart failure and the impact of the management of multiple illnesses, the importance of the doctor-patient relationship, and the role of family and friends, illuminating areas of heart failure care and patient experience that have been insufficiently explored in the previous literature.

## Methods

This study involved secondary analysis of a data-set collected as part of a wider multi-centre investigation, to gain an in-depth insight into the pathways towards, and reasons for, unplanned hospital admissions in patients with heart failure. [20] Primary analysis of the whole data-set had already been conducted, [20] but the data-set comprising patient interviews had not been analysed independently.

For the HoldFAST study, a semi-structured topic guide had been developed by the research team, which allowed respondents to express their experiences of having heart failure along different points in the clinical pathway using a technique referred to as a ‘patient-led ethnography’, which captures views and experiences during important or critical episodes during the patient’s illness journey. Each interview was carried out within the participants’ homes, digitally recorded and transcribed for thematic analysis by the primary researchers. However, shorter ‘debrief’ discussions were held with patients during ‘critical moments’ such as at clinic appointments, GP visits, conversations on the way to or from hospital. The lead researcher (SMc) kept in regular contact with patients who were asked to notify the research team prior to any planned clinic appointments or ‘eventful’ illness episodes that they experienced in order to trigger a visit or telephone discussion by the researcher. This approach provided insights into patients’ naturally evolving illness experiences related to their heart failure journey, and enabled the researchers to record events as they happened, or shortly afterwards. This technique is different from traditional single interviews, as it helped to capture an evolving storyline in the patients’ lives across a period of up to 6 months, to offer a deeper insight into their daily and weekly illness experiences and contacts with health services, particularly around hospital admission.

Ethical approval for the HoldFAST study was granted by NRES Committee South West - Frenchay, Bristol, and local approvals were obtained from Staffordshire Cluster of PCTs Research Management and Governance Office, and the Research & Development Department of University Hospital of North Staffordshire NHS Trust. The original consent form for the HoldFAST study asked the patient to consent to the use of data for further research within the research institutes.

The aim of this secondary analysis was to interrogate a single data-set (the ‘exit interviews) from an independent perspective, in order to critically evaluate the data with the aim of identifying key experiences and impact of living with heart failure and to gain a deeper understanding, and maximise use, of the data [21, 22].

### Data collection

The data comprised anonymised interview transcripts from the participants who had been identified and recruited for the HoldFAST study from a cardiology ward and specialist heart failure ambulatory clinic from three geographical locations within the UK; the Midlands, South Central and the South West of England. Patients with severe or difficult to manage heart failure from the Midlands and South Central and less ‘severe’ patients from a primary care centre from the South West of England were approached. They were given information leaflets and given at least 24 h to consider participating before being contacted by members of the research team. From those willing to participate (*N* = 31) written consent was taken. Nine patients (4 males and 5 females with a mean age = 71.2 years) and three female carers were recruited for the main study. Two patients and three carers also agreed to write diaries. Two other patients (1 male, 1 female) were not recruited into the main HoldFAST study but agreed to participate in single in-depth interviews. The data used for the current analysis were collected over an 11 month period during 2011–2013 and focused on one of the three research sites. The interviews presented in this paper were conducted at the end of the study, (we called these ‘exit interviews’) and encouraged patients to reflect on their illness journey since their diagnosis as well as on their experiences prior to diagnosis. The time of the exit interview from the initial invitation to participate in the study, was up to 6 months.

### Analysis

For this study, a secondary analysis of the interview data was conducted. This involved re-visiting the ten exit interviews and one set of field notes for an interview which wasn’t audio recorded. Following familiarisation with the data-set, each interview was read thoroughly by MF, SMc and CCG and the most prominent themes were identified and agreed. Linking this with the existing literature, a framework was developed by MF, SMc and CCG (see Table 1) to interrogate the data-set.

The initial framework included the questions:

**Table 1:** The questions initially developed to interrogate the interview data

1. What are people’s understanding and perspectives of heart failure?	
2. What are the implications of heart failure?	
3. What is the impact of being diagnosed with heart failure?	
4. How do patients describe heart failure to other people?	

As coding of the first two interview transcripts progressed using the framework (Table 1), and with discussion within the supervisory team, it became apparent there was rich data on the impact of symptoms on everyday life, and narrative about relationships with healthcare practitioners, access to care and the importance of patients’ support networks. Thus, the framework used was modified to include these themes (Table 2) and used interrogate the rest of data-set.

The revised framework included the questions:

**Table 2:** Questions from the revised framework were used to interrogate the data

What are the implications of living with heart failure? - The impact of illness on everyday life - What is the role of family and friends in providing support and sharing responsibility for illness management?	
Relationships with healthcare professionals - How do patients manage multiple illnesses or co-morbidities? - How patients describe their experiences of seeking healthcare?	

High level codes, which highlighted repetitive themes, were identified within the transcripts, using the framework through an iterative method. Regular discussion with the supervisory team (CCG and SM) ensured agreement on coding and that any discrepancies were resolved. These themes had not been previously developed in the primary analysis (as this data-set had not been analysed on it’s own) and thus our analysis provided new insights from the same data set.

## Results

Using the above framework a number of prominent themes will be presented. These are: 1) the impact of illness on everyday life, 2) the role of family and friends in providing support, 3) relationships with health professionals, 4) managing multiple illnesses, and 5) access to healthcare. Each theme is presented and supported by illustrative data, followed by the participant’s unique identifier.

Table 3 gives details of the study participants. Data is given to illustrate each of the themes presented.

**Table 3:** Participant demographics

Respondent	Gender	Age	Interviewed with relative/carer? (Y/N)
1	Female	87	N
2	Female	66	N
3	Female	64	N
4	Male	62	Y
5	Male	67	Y
6	Female	85	Y
7	Male	78	N
8	Male	67	N
9	Female	65	N
10	Male	80	N
11	Female	88	Y

### Implications of living with heart failure

#### Impact of illness on everyday life

Participants described the impact on their lives of living with heart failure symptoms. They described how they experienced the symptoms of heart failure and their impact on everyday routines.

“*Because really you know it’s a condition and sometimes you know, the fluid, obviously my ankles hurt…”* KP8

“*I’d taken the dog for a walk and something started and I thought whatever’s the matter this strange and then I said to my husband, I’m not well*…” KP1

Some participants reported that they had not initially recognises their symptoms as being related to heart failure, and therefore were slow to act upon them at the start of their illness. Following receipt of a diagnosis, participants and their family described heart failure symptoms as a ‘disruption’ to their lives, which made them accept their illness and the limitations of their condition.

“*I remember I didn’t feel as hungry and everything seemed to be sticking you know kind of thing, you know, when you’ve sort of eaten too much…”* KP1

Participants reported a wide range of symptoms, which individually may not self-evidently indicate heart failure. These symptoms limited the patients’ quality of life and impacted on them and their families. Educating patients with heart failure to recognise early symptoms will empower them with the knowledge to identify exacerbations and engage in preventative self-management.

#### Managing multiple illnesses

The majority of respondents stressed the impact of other health problems in combination with their heart failure. They described the difficulty of differentiating the symptoms they were experiencing as a result of the heart failure from their other co-morbidities or side-effects of medications.

“…*but then you don’t know if it’s a cough…so you don’t know whether it’s a coldy-cough or whether it’s something to do with the medication.”* KP6

“..*all the symptoms were there for heart failure but then the symptoms were there like me blood glucose dipped down to two point something so you know it was a mixture of…”* KP3

The main impact of their other illnesses seemed to be the difficulty of managing complex medication regimes.

“*While he’s still messing with the gout medicine he thinks they’ll have to keep me on*…”KP5

“…*of course I was on tablets already for high blood pressure, which I’ve been on for years, and they changed all those”.* KP7

Some respondents reported that they felt that other health conditions they had seemed to be de-prioritised during their treatment for heart failure. This sometimes affected respondents’ feelings towards other healthcare professionals, and they suggested that their co-morbidities were not given equal attention as their heart failure.

“*They were more concerned about the lungs to be honest with you than they were about the heart then…I suppose you go from one of them.”* KP2

“*I was very worried because I thought well I knew I’d got high blood pressure but I thought if they take me off these tablets what will happen you know about my high blood pressure but he said ‘forget all about that, we’ll put you on these others’ which they did*.” KP1

Respondents also discussed the difficulties in managing multiple appointments for their different conditions.

“*I think the 9*^*th*^*of October that’s in my kidneys… Yeah it’s every about four months, six months, just depends what’s going on next week… I was too upset about this cancer to be honest but she thinks, er, I probably won’t get any treatment because I’m too poorly but I’ll have to wait to see what he says on Tuesday”.* KP9

Many of the respondents had multiple illnesses; however they suggested that their heart failure was often prioritised by clinicians, who considered it as having a greater impact on morbidity and mortality.

### The role of family and friends

All of the respondents interviewed discussed their feelings about having or not having a support network of family and/or friends during their illness and the impact this had on them emotionally.

*“But my eldest lad, [name of KP10’s elder son], said you never saw yourself dad, you looked bloody terrible..... that was when they took me… to the accident and emergency”* KP10

*“If [name of KP7’s son] and [name of KP7’s son’s partner] hadn’t had been so good, you know, coming down. They’d come and clean through for me a few times er during that period ‘cause they got on top of me.”* KP7

Some respondents reported family members who took a very proactive role in trying to help participants, either by contacting the health services on their behalf or suggesting decisions for the patient against other doctors’ recommendations.

“*I’ve got a very good friend whose husband is a medical consultant and I did speak to him because I was so concerned and he actually gave me a lot of advice of what to do and what to say to the GP if she didn’t do, erm, what he’d actually advised she should do.*” KP4

KP11s’ daughter repeatedly asked to see the doctor to seek out explanations-(KP11s’ field notes).

Only one respondent identified a lack of immediate family support, and she described a sense of worry and lack of confidence in managing her heart failure:

“*I hope to go shopping but I just feel that I need somebody with me where it’s never bothered me before…but I used to, you know never a care in the world really despite of all my problems.”* KP1

In contrast, the other respondents with strong support networks of family and friends, appeared to accept their diagnosis of heart failure more quickly and described looking to get back to their normal everyday lives building on that support.

*“I’m on top of it again now… I’ve taken charge of it all myself… They cut me lawns for me and that sort of thing, because doing the mowing now – that does kill me ....But as far as the house is concerned, and washing is concerned, I’m doing all me own”* KP7

Family and friends were seen to be key to patients’ recovery.

### Relationships with healthcare professionals

Respondents expressed a spectrum of opinions about the care they had received from their healthcare professionals. They showed appreciation for specialist doctors and specialist heart failure nurses, who were perceived to spend a greater amount of time providing information both to the participants and their families.

“*He’s marvellous… we thought that was really good, because if he spends time with everybody like that then it’s like the old-fashioned doctor, isn’t it really? Instead of just packing you off…*” KP6

“..*but they explained what was going on, which to me is good you know because they can explain things and I won’t worry”* KP10

*“He’s lovely, I always had a kiss and a hug when I go in and out the clinic… Well he’s looked after me for the last six years with Dr L so, er, we know one another”* KP9

However, some respondents were less positive about primary care professionals. A number of respondents reported an apparent delay in diagnosis by their GP, which had negative effects on their relationship.

“*That was while the doctors were saying chest infections… so they weren’t spotting the fluid*.” KP5

“*Oh it’s your asthma, here....he didn’t even examine me....it’s only when my legs started, my ankles started swelling and we insisted*.” KP4

Those respondents, who expressed some negative feelings towards particular clinicians, described how they were wary about future encounters and the confidence the respondents had in those clinicians.

“*he said he wouldn’t go to the doctors because you felt that, you know, if they just give you another inhaler.”* KP4

“*But I wasn’t my normal GP he just left it at that, I always wonder, mind you I don’t think it could have been avoided what happened.*” KP1

Respondents who reported consultations with clinicians who were not their registered primary care doctor, described negative feelings towards the doctor, and attributed any delay in receiving appropriate care to this doctor. Respondents who described such negative encounters with clinicians reported this as a barrier to seeking care in the future.

#### Access to healthcare

Respondents expressed a range of views about the care and support they received from the different healthcare professionals and about access to and interaction with the healthcare system.

Respondents described a lack of pro-active contact from the healthcare system, both from hospitals and primary care, regarding scheduling appointments and the next stages in their care pathway. These respondents described a degree of uncertainty what would happen next and whose responsibility it was to monitor and support them. They expressed uncertainty about whose responsibility it was to initiate communication, when hospitals or primary care failed to send information to the respondents as they had said they would.

“…*oh dear could be 18 months ago something like that…I think the doctors must have sent me but I never heard anything from that at all so I don’t know”.* KP1. This respondent also went on to say, “*the doctor’s surgery perhaps could have shown a bit more support but on the other hand perhaps I should have just rung and you know, so perhaps it’s up to me”.*

*“I went to see, er, Dr E, er, whose first words when I went into the appointment were ‘Why are you here?’ and I said, ‘I haven’t a clue because I don’t know who you are or what you do or anything’, and he said ‘No I don’t know who you are either’*”. KP5

Other respondents, however, described certain healthcare professionals as being exceptionally efficient at facilitating direct access to themselves or other parts of the healthcare system quickly. In these instances, patients expressed appreciation for the effectiveness of that particular doctor or nurse, which led to anticipation of a positive future relationship between the healthcare professional and the patient.

“*‘I remember the radiographer at the hospital said it would be ten days for the results’…sent it straight through....everything marvellous”* KP6

Those respondents who felt that their concerns had been dismissed by particular clinicians, or that they had not been listened to reported a negative impact on the future relationship with those clinicians.

## Discussion

### Summary of results

All participants described the symptoms of heart failure as a disruption to their everyday life and limited their ability to perform routine activities, which led to frustration and a significant loss of confidence, and ultimately caused patients to question their own identity and self-esteem.

Factors which can influence how heart failure may disrupt patients’ lives include the speed and efficiency of a diagnosis and treatment. Many respondents described initial misdiagnoses of their illness, which led to significant delays in the correct treatment being instituted. As a consequence of perceived delays in diagnosis, respondents reported losing confidence in their doctors’ abilities to provide the right care for them, which impacted on future help-seeking. Lack of communication between the healthcare system and the participants led to further confusion and concern.

The majority of respondents mentioned how their family and friends played a role in supporting the management of their illness. Where respondents had some medical expertise in the family, they reported agreeing with their families’ opinion over that of the doctors.

For the one respondent who didn’t have immediate family support, she reported a greater loss of confidence and contrasted her illness journey to that of her husband, where she perceived that had received more support and care. This respondent, along with the others, was interested in returning to ‘normal’ and regaining their independence; her lack of confidence, however, impacted on her ability to self-manage and self-monitor her symptoms.

### Comparison with previous literature

The findings from this study support previous studies conducted on the patient’s experiences of living with heart failure. [23] In addition, our findings support Bury’s description of a ‘biographical disruption’, with symptoms and management leading to lives disrupted from their everyday ‘norm’ [2, 24].

The challenge of managing multiple conditions was described, with patients responding to different exacerbations of their illnesses and prioritising the management of their conditions based on the effect each illness had on their daily lives, and individual clinicians being seen to prioritise the illness which they were expert in treating [8].

The results of this analysis highlight the importance of the doctor-patient relationship, with the findings consistent with those of other studies, as patients described a lack of trust or confidence in doctors that were not their ‘usual’ doctor [15, 16]. As well as doctors ‘knowing’ their patients, the patients had a sense of security ‘knowing’ their doctors, demonstrating the importance of experience and trust between a doctor and their patient, since it provides a gateway to accessing healthcare. Respondents seemed to assign blame to the doctors who were not their ‘usual’ healthcare provider, if anything went wrong, or to contemplate if things would have been done differently if their ‘usual’ doctors had been involved instead.

Poor communication can impact on patient care and outcomes [25]. Respondents described receiving unclear directions about their care pathway and ongoing support from their diagnosing doctors, and were confused about whose responsibility it was to initiate communication between hospital and primary care.

All respondents were positive about the specialist heart failure nurses, who were perceived to have more time to dedicate to patients, for explaining their illness and providing support, which was greatly appreciated by the respondents. This additional time allowed for greater patient education on anticipatory care, which involved identifying symptoms that the participant should look out for, to indicate an exacerbation of their illness. Specialist nurses are thought to have greater opportunities to provide education and support to patients and are able to liaise with different clinicians coordinating care to participants with complex multiple co-morbidities [26]. The results from this study reinforce the need for specialist heart failure nurses in the continued management, education and support of patients.

The findings are consistent with previous qualitative studies reporting that who found that respondents described a sense of burden on those around them, but agreed that it would be worse if they were alone [5]. Some respondents described ‘trying to make the best of it’ and trying as best they could to get back to normality, with the help of their loved ones.

Secondary analysis of the HoldFast data has provided support for results from previous qualitative studies and has highlighted how the difficulties in communication between health professionals across sectors impact on the management of patients as a whole.

### Strengths and limitations

The main strength of this study is that a secondary analysis of previously collected qualitative data was performed. This is ethically sound as secondary analysis offers the opportunity to utilise the rich data from the primary study, from another perspective, thus providing another voice to the perspectives of the participants, who invested considerable time and effort in participating [21].

The study has a number of limitations: only one set of interviews, the ‘exit interviews’, were analysed for this study, which may mean that upon analysis of the whole data set, patients’ perspectives may be reported slightly differently. This may also mean that the transferability of this data to the general population of heart failure patients may be limited. The sample size (*N* = 11) is also relatively small, although category saturation had been achieved in the larger data-set.

### Implications for future practice

This study emphasises the importance of good communication between patients and their HCPs; respondents valued clinicians who took time to elicit their concerns, and recognised the burden of their symptoms and management. This study demonstrated that clinicians need to be more aware of patient co-morbidities and burden of treatment, and the increasing need to liaise with other clinicians to provide care for the whole patient, not just for individual conditions. In addition, further clinician education on recognising a diagnosis of heart failure and being able to confidently communicate this to the patient is required, both in primary and secondary care.

## Conclusions

The results from this study highlight the importance of education both for current health professionals and aspiring medical students on the value of communication, with patients and other members of the multidisciplinary team. Specifically, lessons can be learned on how to manage patients with multiple co-morbidities and to communicate those management plans both with the other health professionals involved with their care and to the patient themselves. Effective communication can influence a patient’s overall perspective of their illness and to turn a diagnosis of heart failure from something that prompts ‘biographical disruption’ into something that a patient can accommodate.
